# Sex-Stratified Gut Microbiota Dysbiosis in Cultured Big-Belly Seahorses During a Mass Mortality Outbreak

**DOI:** 10.3390/cimb48090936

**Published:** 2026-09-13

**Authors:** Hangzhou Guo, Zhibin Sun, Huiyu Xiao, Pengfei Yan, Xin’an Wang, Siqi Wang, Annita Yong Seok-Kian, Aijun Ma

**Affiliations:** 1 State Key Laboratory of Mariculture Biobreeding and Sustainable Goods, Yellow Sea Fisheries Research Institute, Chinese Academy of Fishery Sciences, China-ASEAN Belt and Road Joint Laboratory on Mariculture Technology (Qingdao), Qingdao 266071, China; 18231018539@163.com (H.G.); xiaohy1314@163.com (H.X.); 13234727675@163.com (P.Y.); wangxa@ysfri.ac.cn (X.W.); 18266531907@163.com (S.W.); 2 Graduate School of Chinese Academy of Agricultural Sciences, Beijing 100081, China; 3 Higher Institution Centre of Excellence (HICoE), Institut Marin Borneo, University Malaysia Sabah, Kota Kinabalu 88400, Malaysia; annitay@ums.edu.my

**Keywords:** *Hippocampus abdominalis*, gut microbiota, *Vibrio*, sex differences, mass mortality

## Abstract

Mass mortality outbreaks threaten cultured big-belly seahorses (*Hippocampus abdominalis*), but gut microbial changes associated with disease and host sex remain unclear. We analyzed 36 seahorses from a single aquaculture system, classified as apparently normal males (NMHA), diseased males (EMHA), apparently normal females (NFMHA), and diseased females (EFMHA). Intestinal contents from three individuals were pooled per biological replicate, yielding three pooled replicates per group and 12 microbiome samples in total (effective *n* = 3 per group). The V3–V4 region of the 16S rRNA gene was sequenced, and ASVs were inferred using DADA2 in QIIME 2. α-diversity showed marginal overall group differences, whereas exploratory two-way ANOVA indicated effects of health status and Health × Sex interactions on observed ASVs, Shannon, and Simpson indices. Bray–Curtis PERMANOVA also detected significant effects of health status and the Health × Sex interaction on community structure. Pseudomonadota dominated all groups, while *Vibrio* was strongly enriched in diseased females, reaching 86.94 ± 13.03%. LEfSe identified *Vibrio*/Vibrionaceae as biomarkers of diseased females, whereas Arcobacteraceae and *Litoreibacter* characterized diseased males. PICRUSt2-based prediction suggested higher predicted representation of pathways related to signal transduction, membrane transport, biofilm formation, chemotaxis, and flagellar assembly in diseased groups. Overall, gut microbiota dysbiosis during the outbreak showed sex-stratified patterns, with pronounced *Vibrio* enrichment representing a prominent disease-associated microbial feature, particularly in females.

## 1. Introduction

The big-belly seahorse (*Hippocampus abdominalis*) has both ornamental and economic values. However, populations frequently suffer from mortality events under intensive aquaculture conditions, characterized by a short disease course, rapid death, and atypical clinical signs [1]. Disease outbreaks have recently emerged as a major bottleneck restricting development of the seahorse aquaculture industry. Chen et al. demonstrated that mass mortalities triggered by complex co-infections frequently occurred in large-scale seahorse farms in northern China [2], highlighting the vulnerability of the seahorse gut microbiota under disease stress [3]. Furthermore, modulating seahorses’ intestinal microecology to increase their immunity and gut health has proven to be a key strategy for mitigating mortality risks during the larval rearing stage [4].

Previous studies isolated *Vibrio alginolyticus* and *Vibrio splendidus* from diseased seahorses and reported that *Vibrio tubiashii* could cause major histopathological damage in the lined seahorse (*Hippocampus erectus*) [5,6]. Studies on enteritis in the yellow seahorse (*Hippocampus kuda*) and *Edwardsiella tarda* infection in seahorses further indicated that intestinal diseases were frequently accompanied by epithelial damage and microbiota dysbiosis [7,8]. Additionally, research on *Edwardsiella piscicida* infections and *Vibrio harveyi*-related exposure suggested that disease progression in seahorses often involved coupled shifts in the gut microecology, host response, and disease outcomes [9,10,11]. Consequently, mass mortality events in seahorses are more likely to result from a collective imbalance within the “pathogen–host–microecology” triad, rather than as a linear correspondence between a single pathogen and a single phenotype.

The gut microbiota in fish plays important roles in digestion, nutrient absorption, energy allocation, mucosal barrier maintenance, and immune homeostasis [12,13,14]. Recent reviews further emphasized that the gut microbiota not only impacted nutrient utilization, but was also involved in maintaining health and the energy balance in aquatic animals [15,16]. Studies in seahorses similarly demonstrated that the gut and its associated mucosal microecology were not static, but community compositions varied across different species and habitats [17,18], while the microecological landscape was continually shaped by the host’s reproductive status and environmental exposure [19,20]. Consequently, in the context of mass mortality, merely evaluating if diversity has decreased is insufficient to explain the shifts in host status, and it is more important to ascertain if the community has become dominated by a minority of opportunistic taxa, and if the background symbiotic network has been synchronously weakened.

Host sex has long been underestimated as a biological factor in fish microbiome research. Researchers have observed clear sex-related differences in the gut microbiota in hermaphroditic fish and obscure cultured pufferfish [21,22], and these differences were also associated with changes in reproductive hormones and metabolic states in species such as *Labeo catla* and *Channa argus* [23,24]. From a broader mechanistic perspective, host biological sex and stress–immunity coupling can also influence the stability and functional output of the gut microbiota [23,25]. The unique reproductive physiology and energy allocation patterns of seahorses suggest that it would be interesting to determine if male and female individuals show different patterns of microecological dysbiosis under infection or severe stress conditions.

Vibriosis remains one of the most common opportunistic bacterial diseases in marine aquaculture systems. Reviews of *V. harveyi* and piscine vibriosis highlighted motility, adhesion, biofilm formation, and transport/tolerance systems as recurrent key features during the colonization and pathogenesis of *Vibrio* species [26,27]. Studies of acute *Vibrio* infections in fish indicated that the gut microbiota could also undergo significant structural reorganization within a short period [28]. Based on this, we constructed a 2 × 2 factorial design of “health status (normal/diseased) × sex (male/female)” using individuals from the same aquaculture system and the same cohort. Utilizing 16S rRNA amplicon sequencing and PICRUSt2 functional inference, this study aimed to: (1) quantify the main and interactive effects of disease and sex on α- and β-diversity; (2) identify key taxa and group-specific biomarkers associated with disease segregation, with a focus on determining if *Vibrio* dominance exhibited sex stratification; (3) examine the relationship between community structural shifts and predicted functional potential; and (4) provide testable clues for future validation of sex-stratified microbial indicators associated with seahorse disease outbreaks.

## 2. Materials and Methods

### 2.1. Experimental Animals and Sample Collection

Cultured big-belly seahorses (*H. abdominalis*) were obtained from a commercial aquaculture breeding facility (Weihai Yinze Biological Technology Co., Ltd., Weihai, China). All individuals were aged 7 months and were from the same cohort. All 36 seahorses were co-housed in the same aquaculture tank immediately before sampling, with males and females maintained together. Apparently normal and diseased individuals were sampled on the same day during the same naturally occurring mortality outbreak. Seahorses were collected randomly from a uniform aquaculture tank and categorized based on the clinical phenotypes observed during a mass mortality event. Apparently normal seahorses showed active swimming behavior and rapidly escaped after mild stimulation, with no obvious external abnormalities. Diseased seahorses showed markedly reduced responsiveness and escaped only after strong stimulation, although fin movements remained visible. Diseased individuals also exhibited clinical signs including abdominal distension and erythema around the urogenital pore. These behavioral and gross clinical criteria were used to classify the health status of the sampled seahorses. This study therefore represents a cross-sectional observational design with no repeated or longitudinal sampling.

The seahorses were classified into four groups according to sex and health status: normal males (NMHA), diseased males (EMHA), normal females (NFMHA), and diseased females (EFMHA), with nine individuals in each group. Within each group, the nine seahorses were randomly divided into three non-overlapping pools of three individuals. Equal amounts of intestinal contents from the three individuals in each pool were combined to form one pooled biological sample, yielding three pooled biological replicates per group and 12 pooled samples in total. Pooling was used to obtain composite samples representing group-level microbial profiles during the mortality outbreak while limiting the disproportionate contribution of any single individual to a pooled sample. Each pooled sample, rather than each individual seahorse, was treated as the experimental unit in all microbiome analyses (Table 1). Prior to dissection, seahorses were anesthetized using MS-222 (Sigma-Aldrich, St. Louis, MO, USA) at 150 mg/L for 5 min. The intestinal contents were then collected aseptically, snap-frozen in liquid nitrogen, and stored at −80 °C for DNA extraction. Because this observational study was conducted during a naturally occurring mortality outbreak, no a priori sample-size or power calculation was performed.

### 2.2. DNA Extraction and 16S rRNA Gene Sequencing

Total genomic DNA extraction from the pooled intestinal samples, library construction, and sequencing were performed by a commercial provider (Majorbio Bio-Pharm Technology Co. Ltd., Shanghai, China). The V3–V4 hypervariable region of the bacterial 16S rRNA gene was amplified using the universal primer pair 338F and 806R [29]. The target insertion fragment length was approximately 468 bp. Libraries were prepared using the NEXTFLEX Rapid DNA-Seq Kit (Cat. No. NOVA-5144; Bioo Scientific, Austin, TX, USA). Paired-end sequencing (2 × 300 bp) was performed on an Illumina NextSeq 2000 platform using the NextSeq 1000/2000 P2 XLEAP-SBS Reagent Kit (600 cycles; Cat. No. 20100984; Illumina, San Digeo, CA, USA). No extraction blanks, PCR-negative controls, or mock-community positive controls were included in the sequencing workflow.

### 2.3. Bioinformatics and Statistical Analysis

Raw reads were quality-filtered using fastp v0.23.4 by trimming bases with quality scores below 20 using a 50-bp sliding window; reads shorter than 50 bp or containing ambiguous bases were removed. Paired-end reads were merged using FLASH v1.2.11 with a minimum overlap of 10 bp and a maximum mismatch ratio of 0.2. ASVs were inferred using the DADA2 plugin implemented in QIIME 2 v2024 [30,31]. Taxonomy was assigned using the classify-sklearn Naive Bayes classifier against the SILVA 138.2 16S bacteria reference database with a confidence threshold of 0.7. Core microbiota was defined at the genus level as taxa detected at non-zero abundance in all 12 pooled samples, corresponding to a prevalence of 100%. No additional minimum relative-abundance threshold was applied. Taxonomic nomenclature followed the current bacterial classification, with Pseudomonadota, Bacillota, and Bacteroidota corresponding to the former names Proteobacteria, Firmicutes, and Bacteroidetes, respectively [32]. Sequencing depth was evaluated using the number of effective sequences, rarefaction curves, and Good’s coverage. α-Diversity indices included observed ASVs, Chao1, Faith’s phylogenetic diversity (PD), Shannon index, and Simpson index. Global differences among the four groups were assessed using the Kruskal–Wallis test, with the false discovery rate (FDR) controlled via the Benjamini–Hochberg method. A two-way linear model was also constructed, incorporating health status, sex, and their interaction term, to evaluate interaction effects. Residual normality and homogeneity of variance were assessed using the Shapiro–Wilk and Brown–Forsythe tests, respectively. Given the limited number of pooled biological replicates per factorial cell, a Freedman–Lane permutation-based sensitivity analysis with 99,999 permutations was additionally performed for the Health, Sex, and Health × Sex effects. Benjamini–Hochberg correction was applied within each effect across the examined indices.

β-Diversity was visualized using principal coordinate analysis (PCoA) based on Bray–Curtis and weighted UniFrac distances. Analysis of similarities (ANOSIM) and permutational multivariate analysis of variance (PERMANOVA) were applied to evaluate inter-group structural differences [33]. Differentially abundant taxa at the genus level were initially screened via the Kruskal–Wallis test, followed by pairwise comparisons and linear discriminant analysis effect size (LEfSe; https://huttenhower.sph.harvard.edu/lefse/; accessed on 14 August 2026) using an LDA score threshold of 2.0 to identify group-specific biomarkers [34]. For *Vibrio*, pairwise comparisons among the four groups were performed using the Tukey–Kramer multiple-comparison procedure. Simultaneous 95% confidence intervals and familywise-adjusted *p* values were calculated across all six pairwise comparisons. The relationship between the relative abundance of *Vibrio* and Shannon diversity was evaluated using Spearman’s correlation analysis. All additional statistical analyses and visualizations were performed using R version 4.4.1 (R Foundation for Statistical Computing, Vienna, Austria).

### 2.4. Functional Prediction

The potential functions of the gut microbiota were predicted using PICRUSt2 v2.2.0 based on the representative ASV sequences and abundance tables obtained from 16S rRNA gene sequencing. Initially, the ASV data were pre-processed to filter out non-bacterial sequences and low-abundance features, and functional annotations were subsequently conducted based on the normalized abundance data. The representative ASV sequences were then aligned and placed into a reference phylogenetic tree to infer the gene family composition for each ASV, followed by correction for 16S rRNA gene copy number.

The predicted gene families were further annotated against the Kyoto Encyclopedia of Genes and Genomes (KEGG) Orthology (KO) database to generate a predicted functional abundance matrix for each sample, which was subsequently summarized at different functional hierarchical levels. Differences in predicted functional profiles among groups were analyzed based on these abundance data. Statistical methods were used to compare the predicted relative abundance of functional pathways among groups, followed by FDR correction for multiple testing to identify significantly different predicted functional features. These predicted functional features were visualized using bar charts and heatmaps. All PICRUSt2-derived functional profiles were interpreted as predicted functional potential rather than direct measurements of microbial gene content, gene expression, or metabolic activity.

## 3. Results

### 3.1. Sequencing Output and Asv Inference

**Figure 1:**
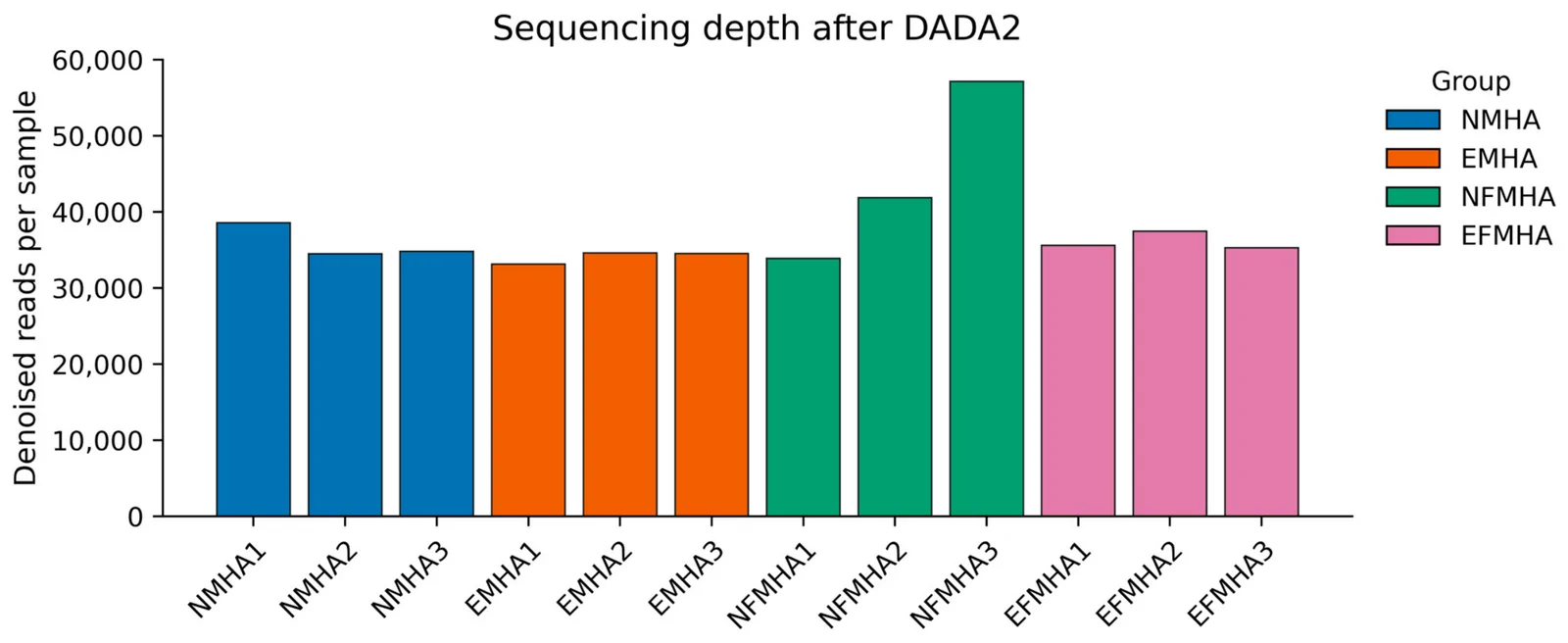
Effective sequence reads of the samples after denoising. The bar plot shows the number of denoised reads retained for each pooled intestinal sample. The *x*-axis represents individual samples from the four groups, including normal males (NMHA), diseased males (EMHA), normal females (NFMHA), and diseased females (EFMHA), while the *y*-axis represents the number of effective sequences per sample. Different colors indicate different experimental groups. All samples retained sufficient sequencing depth after quality control, supporting the reliability of subsequent ASV inference, diversity estimation, and community composition analysis.

**Figure 2:**
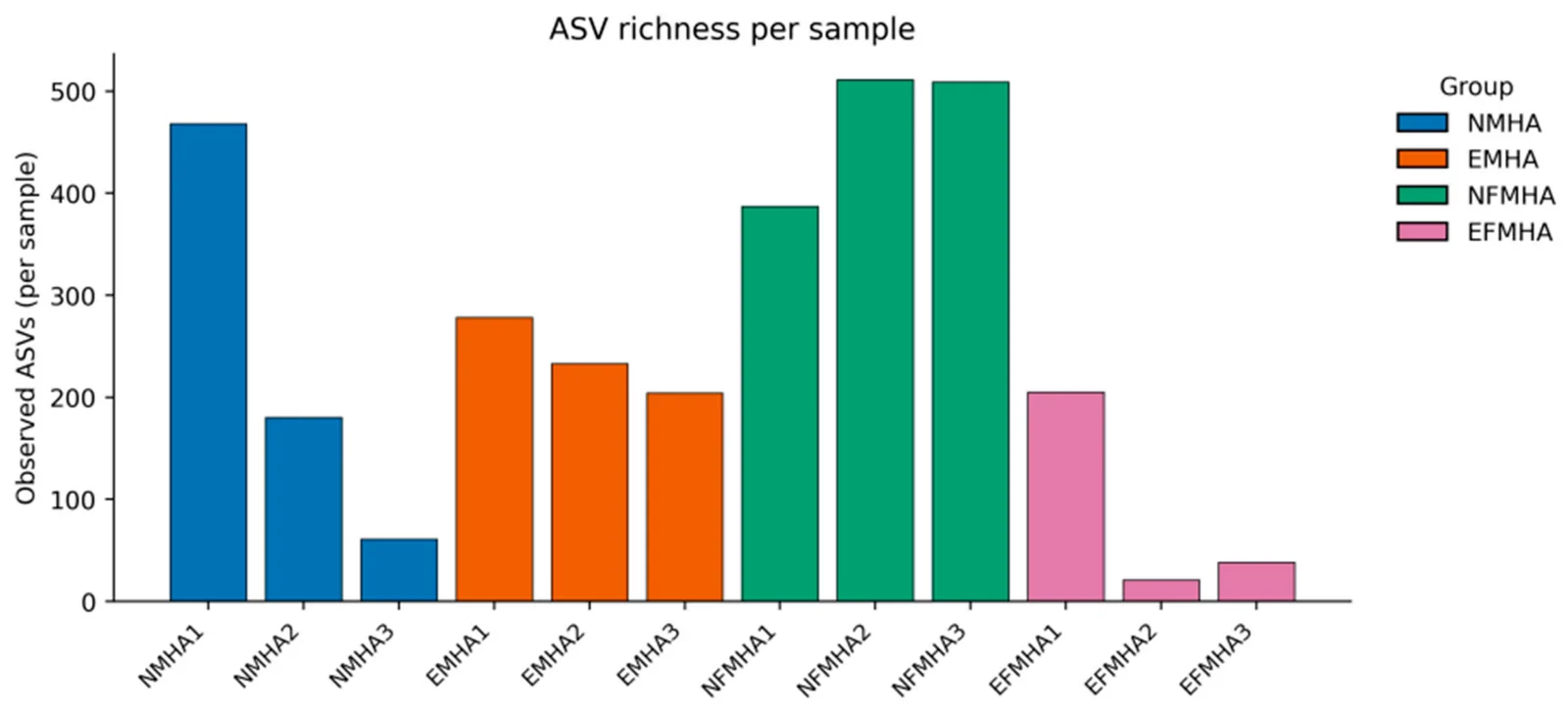
Observed amplicon sequence variant (ASV) richness across the samples. The bar plot illustrates the number of observed amplicon sequence variants (ASVs) detected in each sample. The *x*-axis represents individual pooled samples, and the *y*-axis indicates the observed ASV richness. Samples from the healthy female group (NFMHA) generally showed higher ASV richness, whereas the diseased female group (EFMHA) exhibited a marked reduction in ASV number. This pattern suggests that disease-associated gut microbial dysbiosis was accompanied by a pronounced loss of microbial richness, particularly in female seahorses.

**Figure 3:**
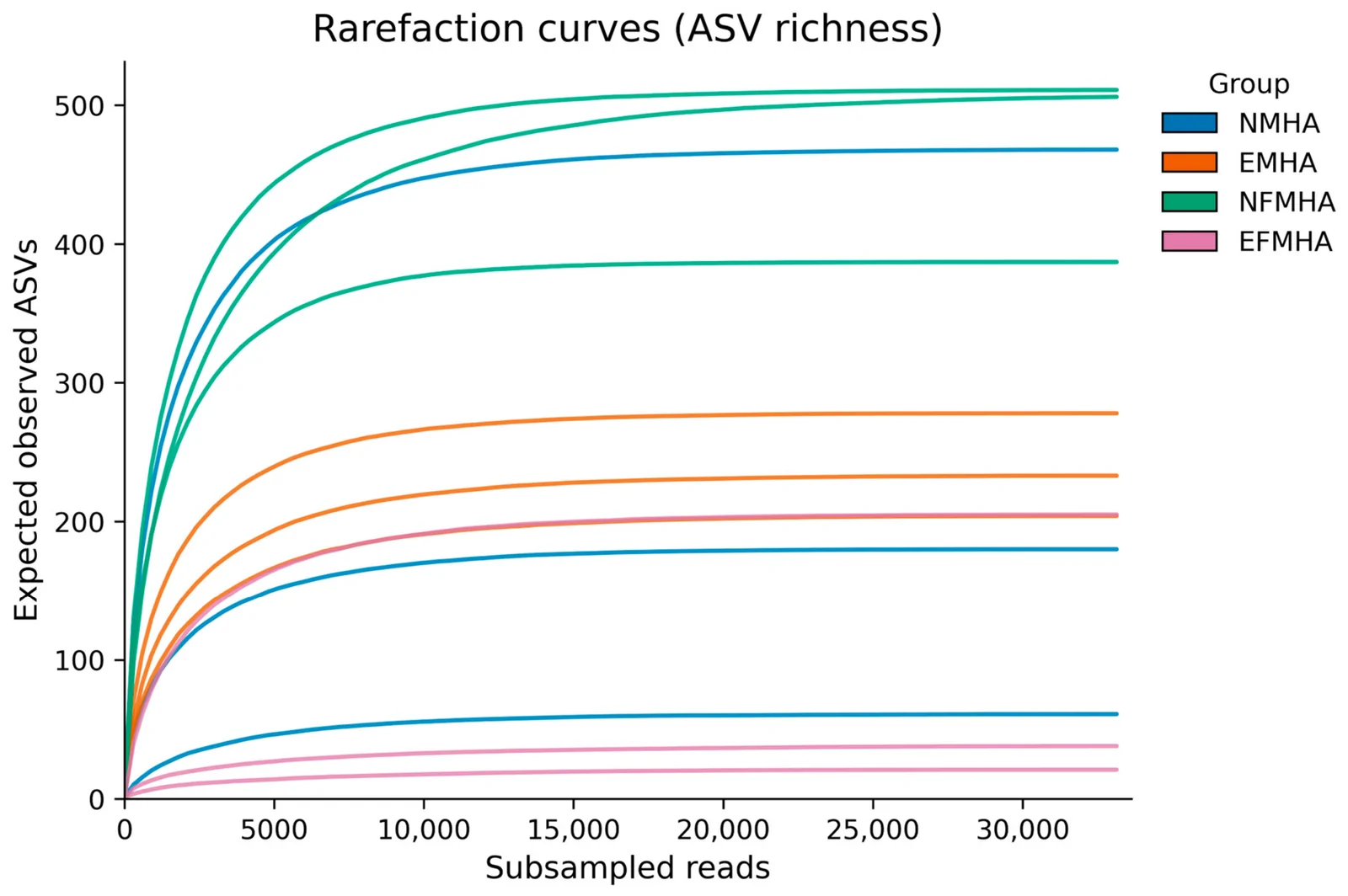
Rarefaction curves illustrating the sufficiency of sequencing depth for each sample. The rarefaction curves show the relationship between sequencing depth and the expected number of observed ASVs. The *x*-axis represents the number of subsampled reads, and the *y*-axis represents the expected ASV richness. Each curve corresponds to one sample, with colors indicating experimental groups. Most curves gradually approached a plateau, indicating that the sequencing depth was sufficient to capture the majority of bacterial diversity in the intestinal samples. The consistently lower curves in the EFMHA group further support the occurrence of reduced microbial richness in diseased females rather than insufficient sequencing depth.

A total of 603,828 raw paired-end reads, encompassing 181,148,400 base pairs (bp), were obtained from the 12 pooled intestinal samples. After denoising, 33,140–57,168 effective sequences were retained per sample (mean = 37,631 ± 6608). The number of inferred ASVs ranged from 21–511 per sample, with an average of 257.9 ± 176.8. The rarefaction curves generally tended to reach a plateau, and the Good’s coverage for all samples was >0.999, indicating that the current sequencing depth was sufficient to capture the vast majority of bacterial diversity within the samples. The sequencing output, observed ASV richness, and rarefaction curves are presented in Figure 1, Figure 2, and Figure 3, respectively.

### 3.2. A-Diversity Analysis

**Figure 4:**
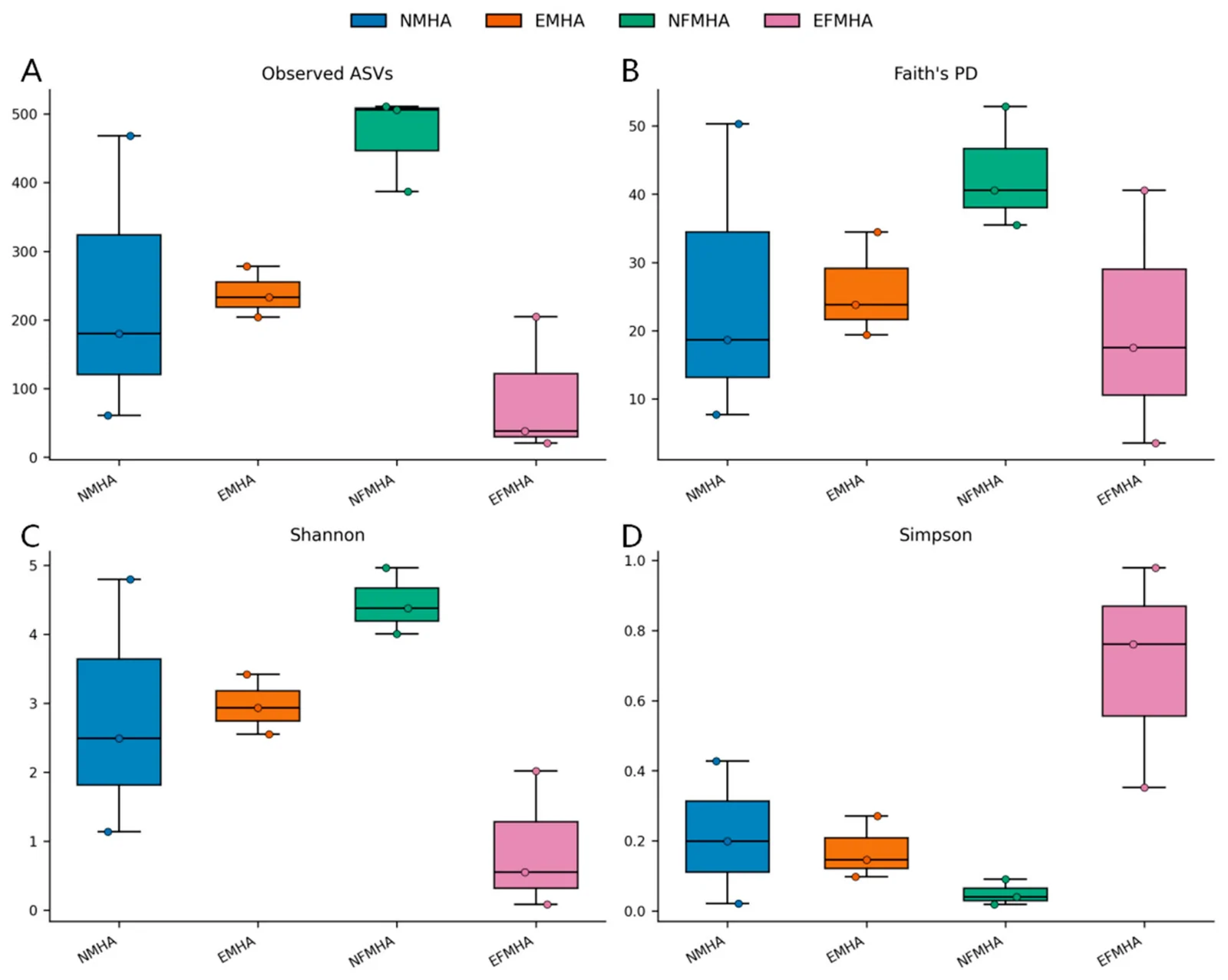
Boxplots of α-diversity indices across the four groups. (**A**) Observed ASVs represent microbial richness; (**B**) Faith’s phylogenetic diversity (PD) reflects phylogenetic breadth; (**C**) Shannon index represents both richness and evenness; and (**D**) Simpson index reflects the degree of community dominance under the index used in this study. Boxplots display the distribution of each α-diversity index among NMHA, EMHA, NFMHA, and EFMHA groups. The NFMHA group showed relatively high richness and diversity, whereas the EFMHA group exhibited decreased Observed ASVs, Faith’s PD, and Shannon values, together with an increased Simpson index. These descriptive patterns suggest lower microbial richness and evenness and greater dominance in diseased females; inferential results are reported in the text and interpreted cautiously because *n* = 3 pooled samples per group.

α-Diversity indices, including observed ASVs, Faith’s PD, and Shannon and Simpson indices, were compared among the four groups (NMHA, EMHA, NFMHA, and EFMHA). The Kruskal–Wallis test revealed marginal differences among the groups for observed ASVs (H = 7.00, *p* = 0.0719, q = 0.0959) and Shannon (H = 7.46, *p* = 0.0586, q = 0.0959) and Simpson indices (H = 7.41, *p* = 0.0599, q = 0.0959), but no significant difference in Faith’s PD (H = 3.31, *p* = 0.3466, q = 0.3466). Pairwise comparisons with Holm’s correction did not reach statistical significance. The distributions of the α-diversity indices across the four groups are shown in Figure 4.

Regarding the within-group distribution, differences were mainly observed in diseased female individuals. The NFMHA group exhibited the highest levels of community richness and diversity, with observed ASVs, Faith’s PD, and Shannon index values of 468.00 ± 70.19, 42.97 ± 8.95, and 4.45 ± 0.48, respectively. These values were notably decreased in the EFMHA group to 88.00 ± 101.68, 20.54 ± 18.70, and 0.89 ± 1.01, respectively. Simultaneously, the Simpson index in the EFMHA group increased to 0.697 ± 0.318, compared with 0.050 ± 0.037 in the NFMHA group, indicating a decrease in community evenness and an increase in the proportion of dominant taxa in diseased females. In contrast, differences between apparently normal and diseased male seahorses were relatively limited. The observed ASVs for the NMHA and EMHA groups were 236.33 ± 209.27 and 238.33 ± 37.29, Faith’s PD values were 25.55 ± 21.81 and 25.88 ± 7.75, and the Shannon indices were 2.81 ± 1.85 and 2.97 ± 0.44, respectively. These descriptive patterns suggest that disease-associated α-diversity changes may have differed between sexes during this outbreak.

Further two-way analysis of variance (ANOVA) indicated significant effects of health status on observed ASVs (F = 7.091, *p* = 0.0287), Shannon (F = 7.158, *p* = 0.0281), and Simpson indices (F = 7.169, *p* = 0.0280), but not on Faith’s PD (F = 1.495, *p* = 0.2563). The main effect of sex was not significant for any of the four indices (*p* > 0.05). Significant Health × Sex interaction effects were detected for observed ASVs (F = 7.242, *p* = 0.0274), Shannon (F = 8.582, *p* = 0.0190), and Simpson indices (F = 9.449, *p* = 0.0153), but not for Faith’s PD (F = 1.586, *p* = 0.2434). Diagnostic evaluation of the ANOVA models did not detect significant violations of residual normality or homogeneity of variance for the α-diversity indices examined. In addition, a Freedman–Lane permutation-based sensitivity analysis with 99,999 permutations retained the Health and Health × Sex effects after Benjamini–Hochberg correction (q ≤ 0.0481), providing complementary support for these factorial effects.

Nevertheless, given the limited number of pooled biological replicates per factorial cell (*n* = 3), together with the non-significant FDR-corrected four-group Kruskal–Wallis results (q ≈ 0.096) and non-significant pairwise comparisons, these α-diversity findings should be regarded as preliminary rather than conclusive. The observed patterns suggest that disease-associated changes in microbial richness and evenness may differ between sexes, but the interaction effects should be interpreted cautiously. The corresponding two-way ANOVA results are summarized in Table 2.

### 3.3. B-Diversity Analysis

**Figure 5:**
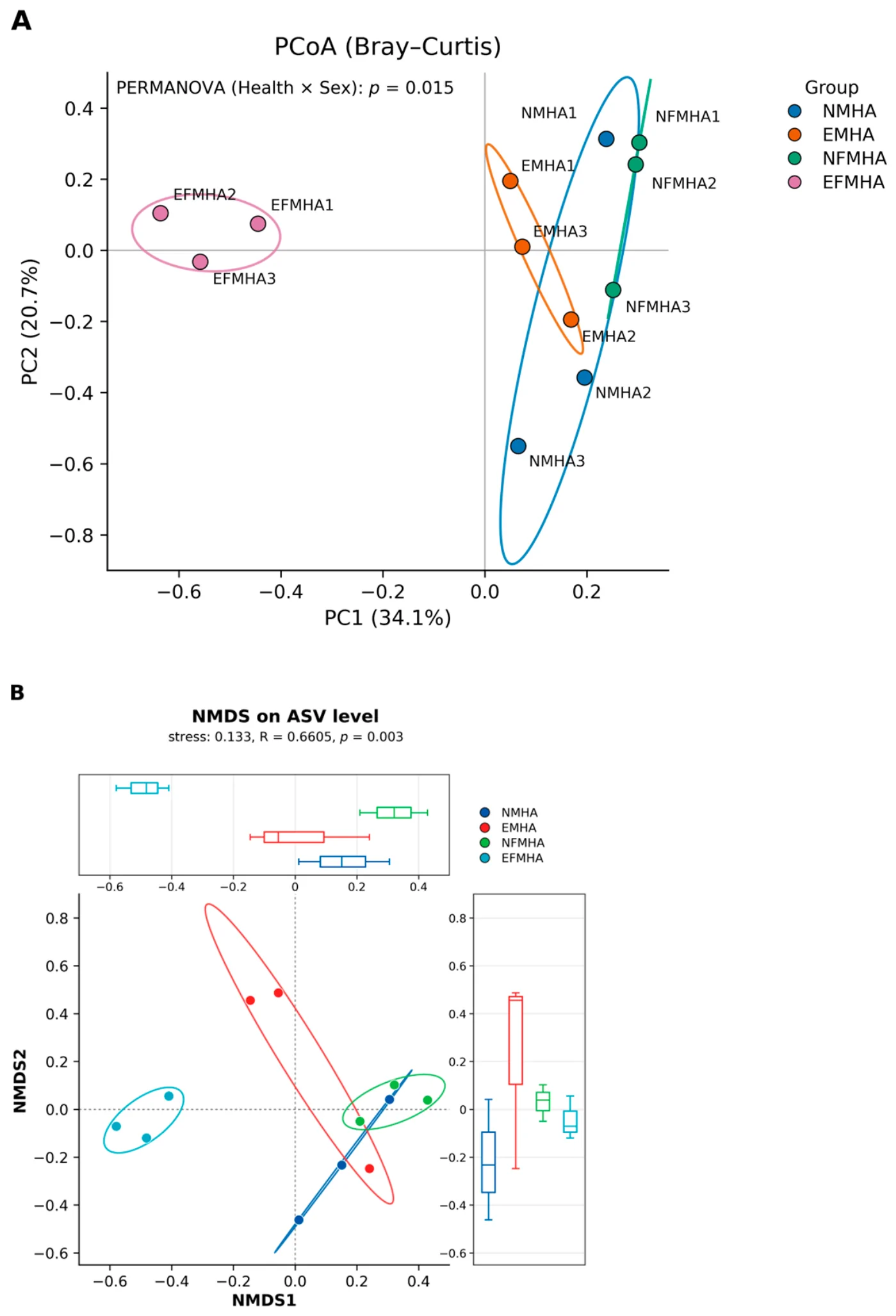
β-diversity patterns of gut microbiota among the four groups based on Bray–Curtis distance. (**A**) Principal coordinate analysis (PCoA) plot based on Bray–Curtis distance. The *x*- and *y*-axes represent the first two principal coordinates, explaining 34.1% and 20.7% of total community variation, respectively. Each point represents one pooled intestinal sample, and colors indicate different experimental groups. (**B**) Non-metric multidimensional scaling (NMDS) plot based on ASV-level community composition. The stress value of 0.133 indicates an acceptable, though not optimal, ordination fit. Both ordination analyses show that the EFMHA group was clearly separated from the other groups and formed a relatively compact cluster. This ordination pattern was associated with pronounced restructuring of gut microbial community composition, particularly in diseased females.

Community structures were compared at the ASV level based on the Bray–Curtis distance. PCoA revealed that the first two axes explained 34.1% (PC1) and 20.7% (PC2) of the total variation, respectively. The four groups exhibited a clear separation trend within the ordination space. Notably, the diseased female group (EFMHA) deviated distinctly from the other groups along PC1, forming a highly compact cluster. The remaining three groups (NMHA, NFMHA, and EMHA) were primarily distributed on the opposite side of PC1, positioned close to each other but distinguishable (Figure 5A). Consistent with the PCoA results, non-metric multidimensional scaling (NMDS) ordination also demonstrated structural differentiation among the four groups in two-dimensional space, with a stress value of 0.133, indicating an acceptable, though not optimal, ordination fit. The EFMHA group consistently formed a distinct cluster and was clearly separated from the other groups in the ordination space (Figure 5B).

ANOSIM indicated strong inter-group differences (R = 0.6605, *p* = 0.003, 999 permutations), suggesting significant differentiation in the overall community composition among the four groups. A global PERMANOVA test using the four groups as a unified factor also reached significance (*p* = 0.015). Upon further decomposition using a two-way model incorporating health status (Health), sex (Sex), and their interaction (Health × Sex), the PERMANOVA results revealed a significant main effect of Health (F = 2.299, *p* = 0.0252, R^2^ = 0.154) and a significant interaction effect of Health × Sex (F = 3.219, *p* = 0.0037, R^2^ = 0.215). However, the main effect of Sex was not statistically significant (F = 1.436, *p* = 0.2026, R^2^ = 0.096). Multivariate dispersion did not differ among groups (BETADISPER, F = 1.1478, *p* = 0.394; 999 permutations). The corresponding two-way PERMANOVA results are summarized in Table 3.

### 3.4. Community Composition and Overall Taxonomic Patterns

The integration of taxonomic profiles at the phylum and genus levels revealed a pronounced remodeling of the gut microbial structure across different health statuses and sexes, which corroborated the observed shifts in α- and β-diversity.

At the phylum level, Pseudomonadota was the predominant taxon across all four groups; however, community composition varied markedly among groups. The apparently normal groups (NMHA and NFMHA) showed a more diverse taxonomic profile, including substantial proportions of Bacillota, Bacteroidota, and other low-abundance phyla. In contrast, the relative abundance of Pseudomonadota further increased and became overwhelmingly dominant in the diseased female group (EFMHA). This trend toward community simplification was consistent with the reduced α-diversity observed in this group, suggesting increased dominance of a limited number of bacterial lineages and reduced community complexity in diseased females.

Differences were even more striking at the genus level. Diseased groups generally exhibited an expansion of *Vibrio*, with the most intense enrichment observed in the EFMHA group, where some samples exhibited pronounced *Vibrio* dominance. Statistically, the relative abundance of *Vibrio* was significantly higher in the EFMHA group than in the NFMHA group (86.94% ± 13.03% (mean ± standard deviation) vs. 1.75% ± 1.54%). A similar upward trend was observed in males, with *Vibrio* increasing from 2.18% ± 3.42% in the NMHA group to 33.19% ± 10.14% in the EMHA group.

**Figure 6:**
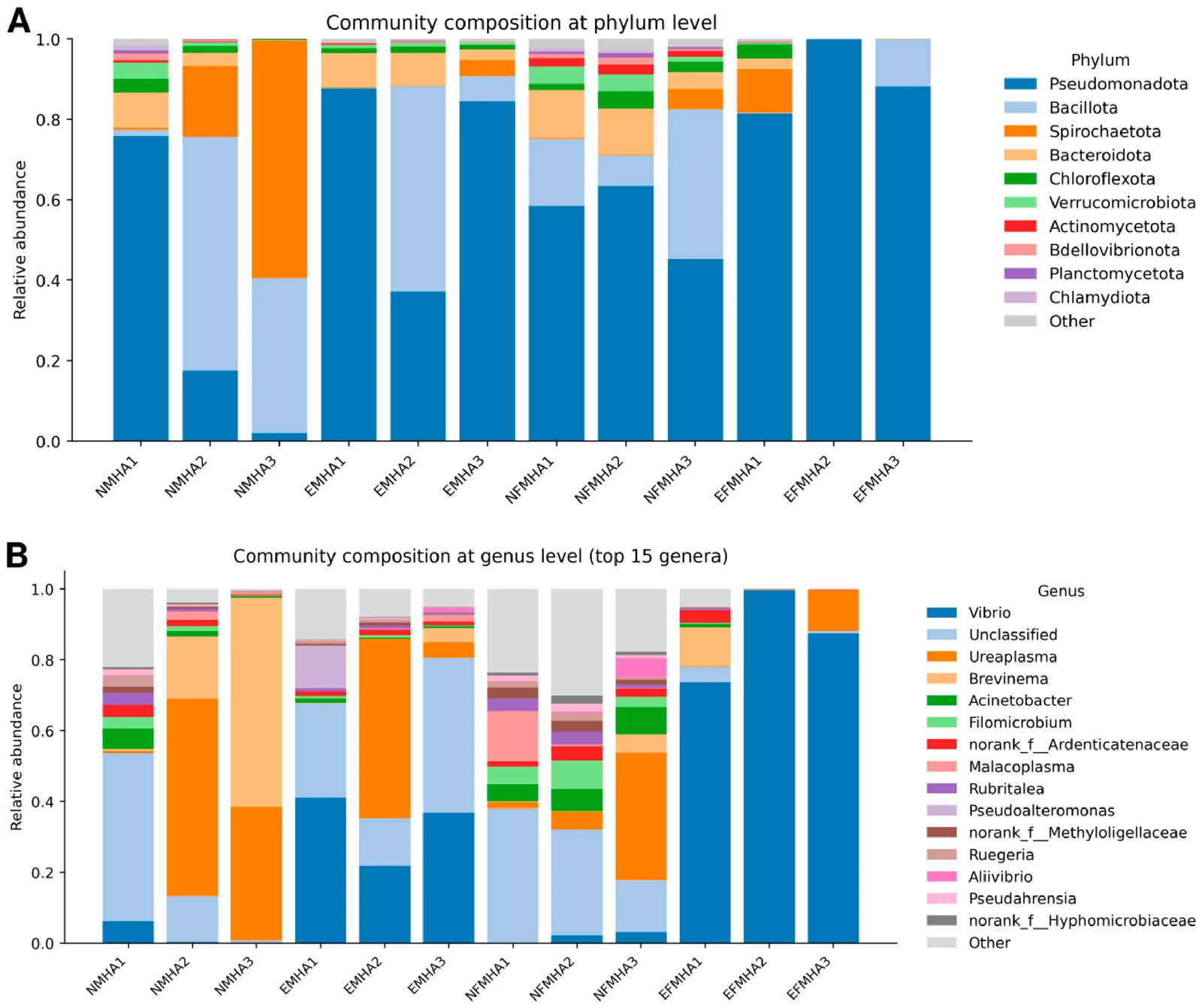
Relative abundance of gut microbiota at the phylum and genus levels across the four groups. (**A**) Relative abundance of dominant bacterial phyla in each sample. The *x*-axis represents individual samples, and the *y*-axis represents relative abundance. Different colors indicate different phyla. Pseudomonadota was the dominant phylum across all groups, but its dominance was especially pronounced in diseased females. (**B**) Relative abundance of the top 15 bacterial genera in each sample. At the genus level, *Vibrio* showed a marked expansion in diseased groups, with the strongest enrichment observed in the EFMHA group. These results indicate that disease-associated gut dysbiosis was characterized not only by changes in overall diversity but also by a shift toward a simplified community dominated by opportunistic taxa, particularly *Vibrio*.

Overall, the taxonomic composition, consistent with the α- and β-diversity results, showed more pronounced disease-associated microbial differences in females during this outbreak. The gut community transitioned from a relatively complex and balanced state to a low-diversity structure dominated by opportunistic taxa, particularly *Vibrio*. The phylum- and genus-level community composition profiles are shown in Figure 6A,B, respectively.

### 3.5. Differential Taxa and Lefse Biomarkers Revealed Sex-Stratified Disease Signatures

To identify the key taxa driving community segregation among groups with different health statuses and sexes, we screened for differentially abundant taxa at the genus level and utilized LEfSe to delineate group-specific biomarkers.

The Kruskal–Wallis test identified 10 genera with nominal *p* < 0.05, including *Vibrio* (*p* = 0.0249), *Filomicrobium* (*p* = 0.0487), *Burkholderia-Caballeronia-Paraburkholderia* (*p* = 0.0324), *Methylobacterium* (*p* = 0.0493), *Breoghania* (*p* = 0.0260), *Litoreibacter* (*p* = 0.0257), *Arcobacter* (*p* = 0.0422), and several unclassified taxa. However, none remained significant after Benjamini–Hochberg correction (lowest q = 0.2798). These genera are therefore presented as exploratory nominal signals rather than FDR-significant differences.

Given the pronounced dominance of *Vibrio* observed in the community composition analysis, Tukey–Kramer pairwise comparisons were performed among the four groups, with simultaneous 95% confidence intervals and familywise-adjusted *p* values calculated across all six pairwise comparisons. The relative abundance of *Vibrio* in the EFMHA group was significantly higher than that in the NMHA, EMHA, and NFMHA groups (all familywise-adjusted *p* < 0.001). EMHA also showed significantly higher *Vibrio* abundance than NMHA and NFMHA (both familywise-adjusted *p* < 0.01). Conversely, no significant difference was detected between NMHA and NFMHA (familywise-adjusted *p* > 0.1). Given the limited number of pooled biological replicates per group (*n* = 3), these pairwise results should be regarded as exploratory. These results indicate that *Vibrio* enrichment was primarily associated with the diseased groups and was more pronounced in females.

LEfSe analysis further refined the group-specific biomarkers from the perspective of statistical significance coupled with biological relevance (LDA score). Diseased females (EFMHA) were significantly enriched in *Vibrio*, Vibrionaceae, Enterobacterales, and Gammaproteobacteria, reflecting a dysbiotic profile characterized by enrichment of *Vibrio* and several other bacterial taxa. In contrast, diseased males (EMHA) were primarily characterized by biomarkers such as Arcobacteraceae, *Arcobacter*, and *Litoreibacter*. Meanwhile, apparently normal females (NFMHA) were enriched in multiple taxa associated with Hyphomicrobiaceae, Microtrichaceae, and Oligoflexales/Oligoflexaceae (displaying high LDA scores), indicating a distinct and phylogenetically diverse taxonomic profile in the apparently normal female gut.

**Figure 7:**
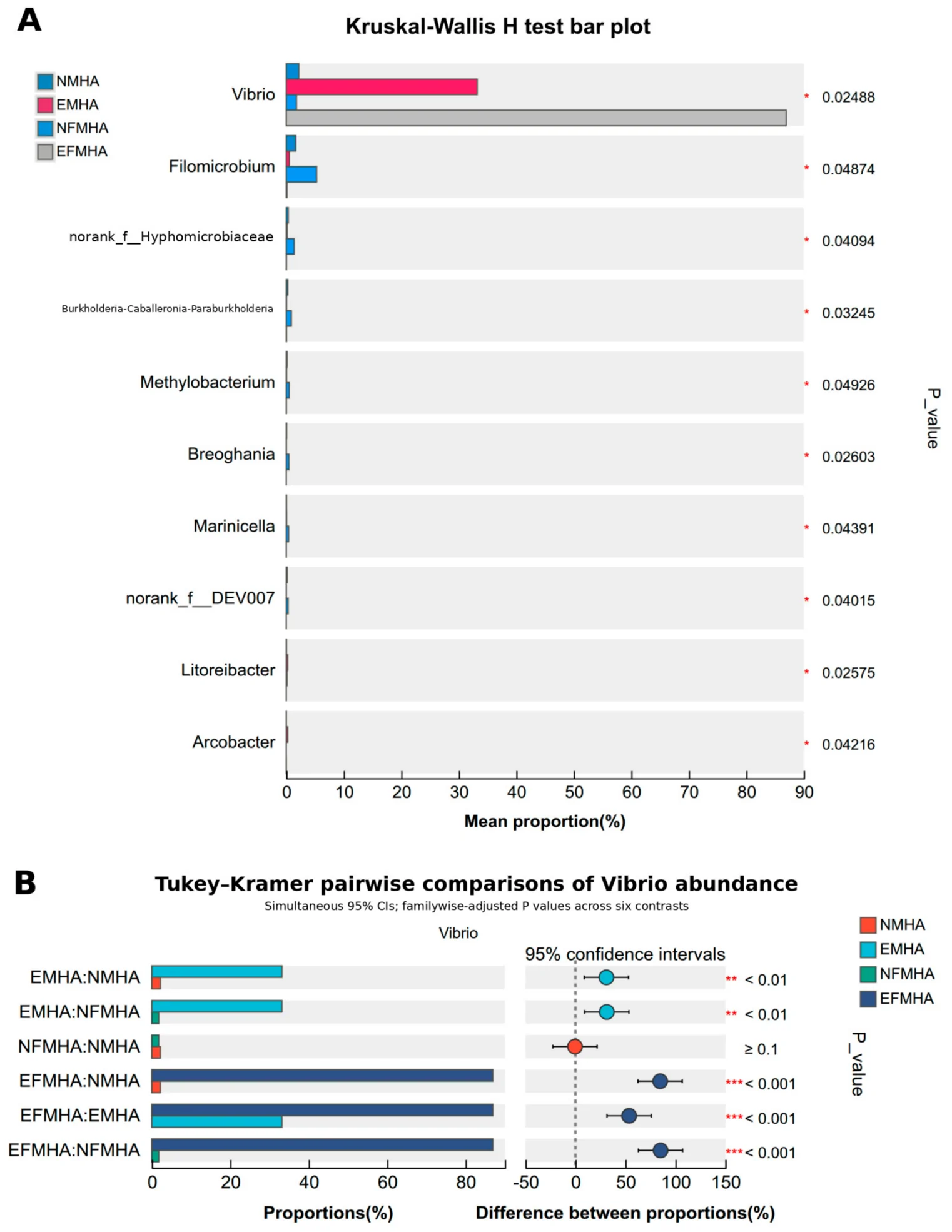

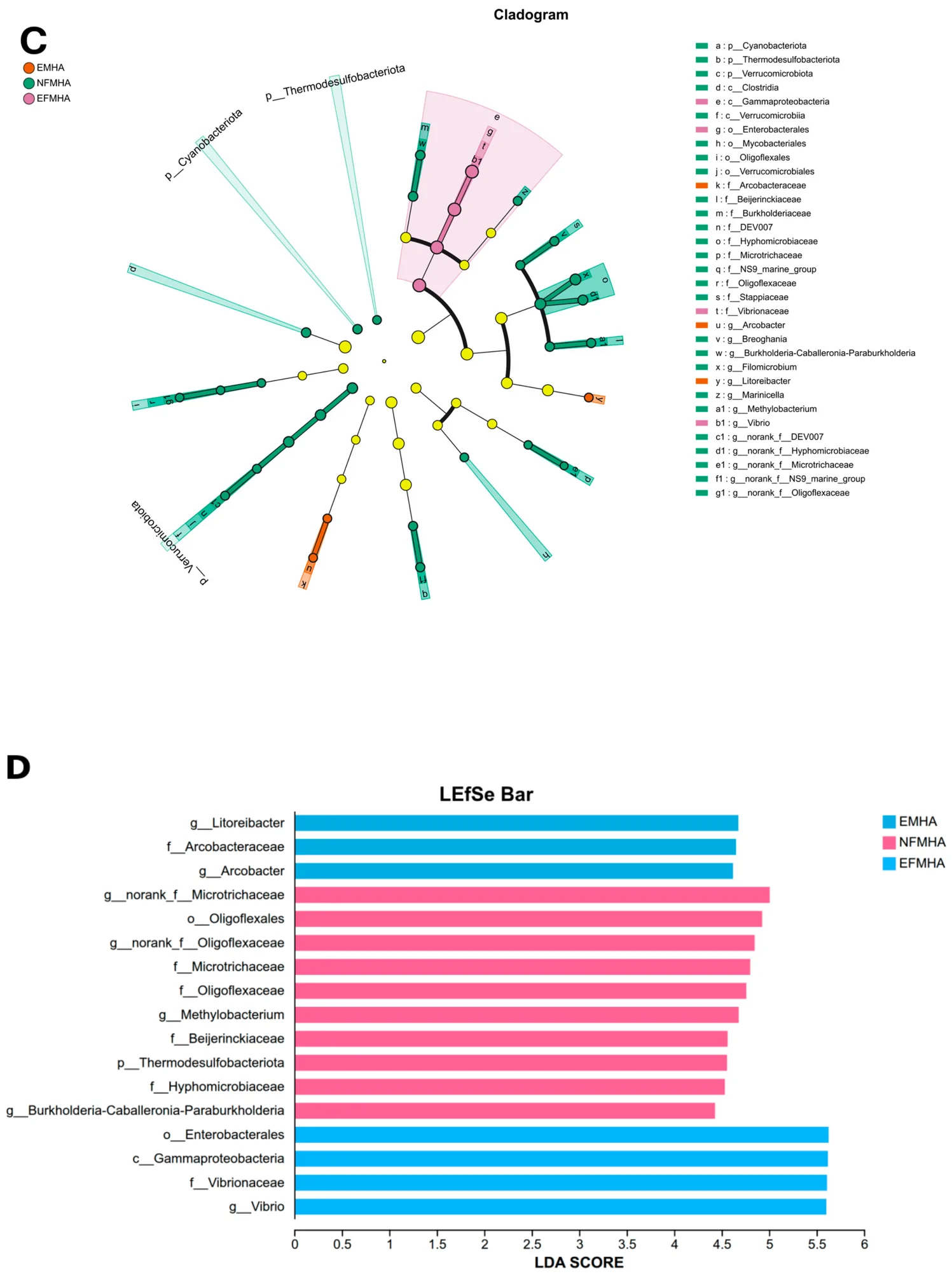
Differential taxa and biomarker analysis revealing sex-stratified disease-associated microbial signatures. (**A**) Genera with nominal Kruskal–Wallis *p* < 0.05 among the four groups. None remained significant after Benjamini–Hochberg correction (lowest q = 0.2798). The *x*-axis represents mean relative abundance, and colors indicate experimental groups. * indicates a nominal Kruskal–Wallis *p* < 0.05 (**B**) Tukey–Kramer pairwise comparisons of *Vibrio* relative abundance among the four groups, showing simultaneous 95% confidence intervals and familywise-adjusted *p* values for all six pairwise comparisons. ** and *** indicate Tukey–Kramer familywise-adjusted *p* < 0.01 and *p* < 0.001, respectively. (**C**) LEfSe cladogram showing the phylogenetic distribution of group-specific microbial biomarkers. Orange, green, and magenta indicate taxa enriched in the EMHA, NFMHA, and EFMHA groups, respectively, whereas yellow indicates taxa without significant group-specific enrichment. (**D**) LDA score bar plot of discriminative taxa identified by LEfSe. Only EMHA, NFMHA, and EFMHA are shown in panels C and D because no NMHA-enriched taxon met the applied LEfSe criteria. Higher LDA scores indicate stronger contributions to group separation. *Vibrio* and Vibrionaceae characterized diseased females, whereas Arcobacteraceae, *Arcobacter*, and *Litoreibacter* characterized diseased males. All taxon-level results should be interpreted as exploratory given the limited biological replication.

Overall, the nominal genus-level screening and LEfSe analysis suggested sex-stratified disease-associated patterns, with *Vibrio* enrichment observed in both diseased groups and most pronounced in diseased females. Given the limited replication and the absence of FDR-significant genus-level Kruskal–Wallis results, these taxon-level patterns should be considered exploratory. The genus-level screening, *Vibrio* pairwise comparisons, LEfSe cladogram, and LDA scores are presented in Figure 7A–D.

### 3.6. Predicted Functional Potential of the Gut Bacterial Community

Functional inference based on PICRUSt2 indicated that the predicted functional profiles of the four groups were primarily represented by categories related to basal metabolism and environmental information processing. At KEGG Pathway Level 2, categories such as carbohydrate metabolism, amino acid metabolism, energy metabolism, membrane transport, signal transduction, and replication and repair showed relatively high predicted representation across all groups. These predictions suggested that the inferred functional potential of the gut microbiota was mainly associated with substrate utilization, substance transport, and basic cellular processes.

Regarding disease-associated differences, the predicted functional profiles showed sex-stratified patterns. Compared with healthy females (NFMHA), diseased females (EFMHA) showed higher predicted relative abundances of Level 2 categories associated with cell motility (approximately 1.71-fold), signal transduction (approximately 1.58-fold), membrane transport (approximately 1.31-fold), and cellular community–prokaryotes (approximately 1.49-fold), whereas the predicted relative abundance of xenobiotics biodegradation and metabolism was lower (approximately 0.50-fold). These patterns suggest potential differences in functional capacity associated with environmental sensing, motility/colonization, and substance transport. In males, the EMHA group similarly showed higher predicted relative abundances of signal transduction (approximately 1.45-fold) and membrane transport (approximately 1.29-fold), whereas translation (approximately 0.64-fold) and replication and repair (approximately 0.79-fold) showed lower predicted relative abundances than in NMHA.

At KEGG Pathway Level 3, predicted differences were mainly observed in pathways associated with microbial adaptation and host interaction. The predicted relative abundance of the phosphotransferase system was approximately 5.14-fold and 4.15-fold higher in diseased females and males, respectively. The pathway annotated as biofilm formation–*V. cholerae* also showed higher predicted representation in both diseased groups (approximately 3.89-fold in females and 4.66-fold in males). Similarly, the predicted relative abundances of the two-component system (approximately 1.73-fold in females and 1.62-fold in males), cationic antimicrobial peptide resistance (approximately 1.79-fold and 2.09-fold), and ABC transporters (approximately 1.09-fold and 1.20-fold) were higher in the diseased groups.

**Figure 8:**
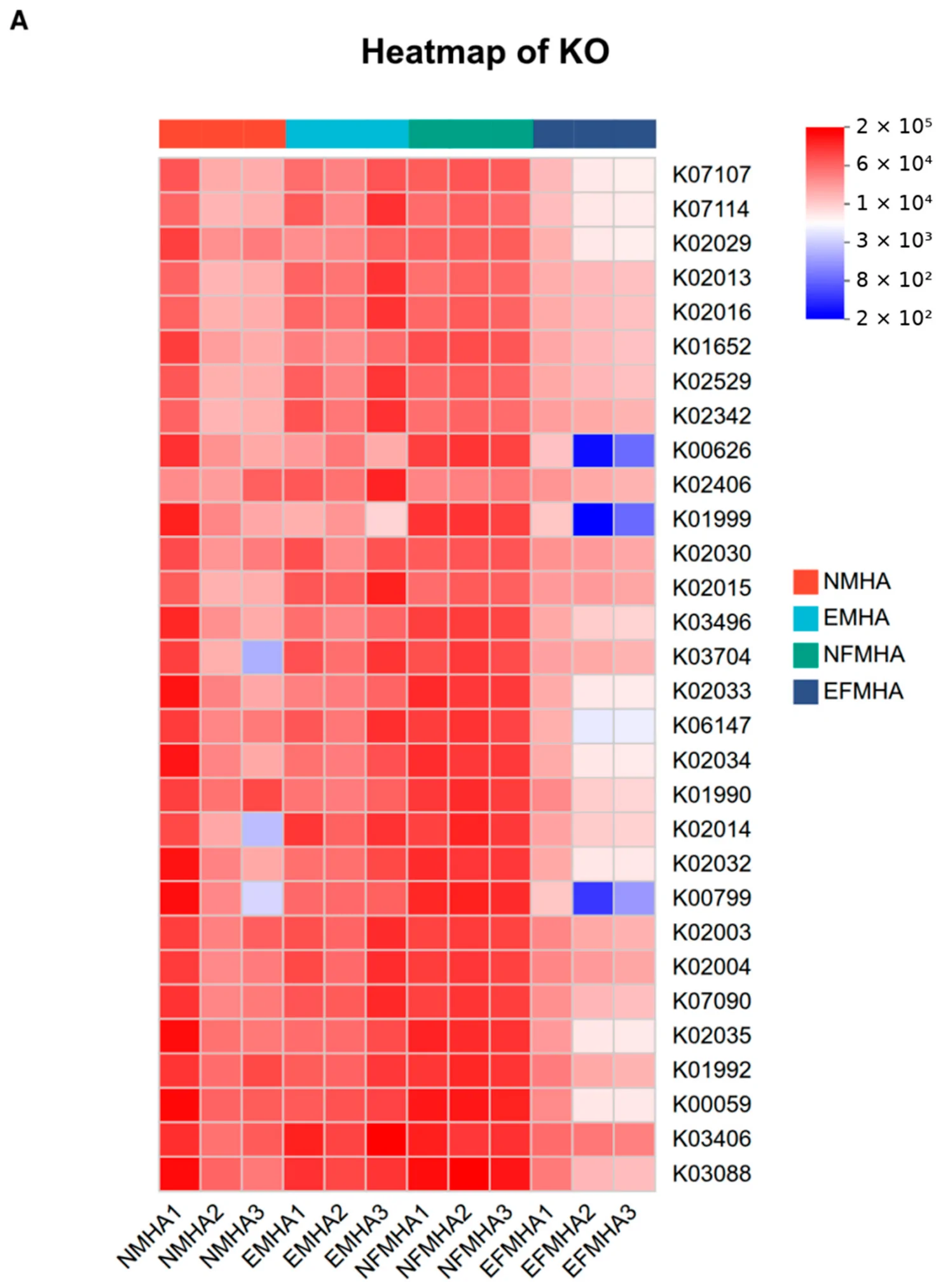

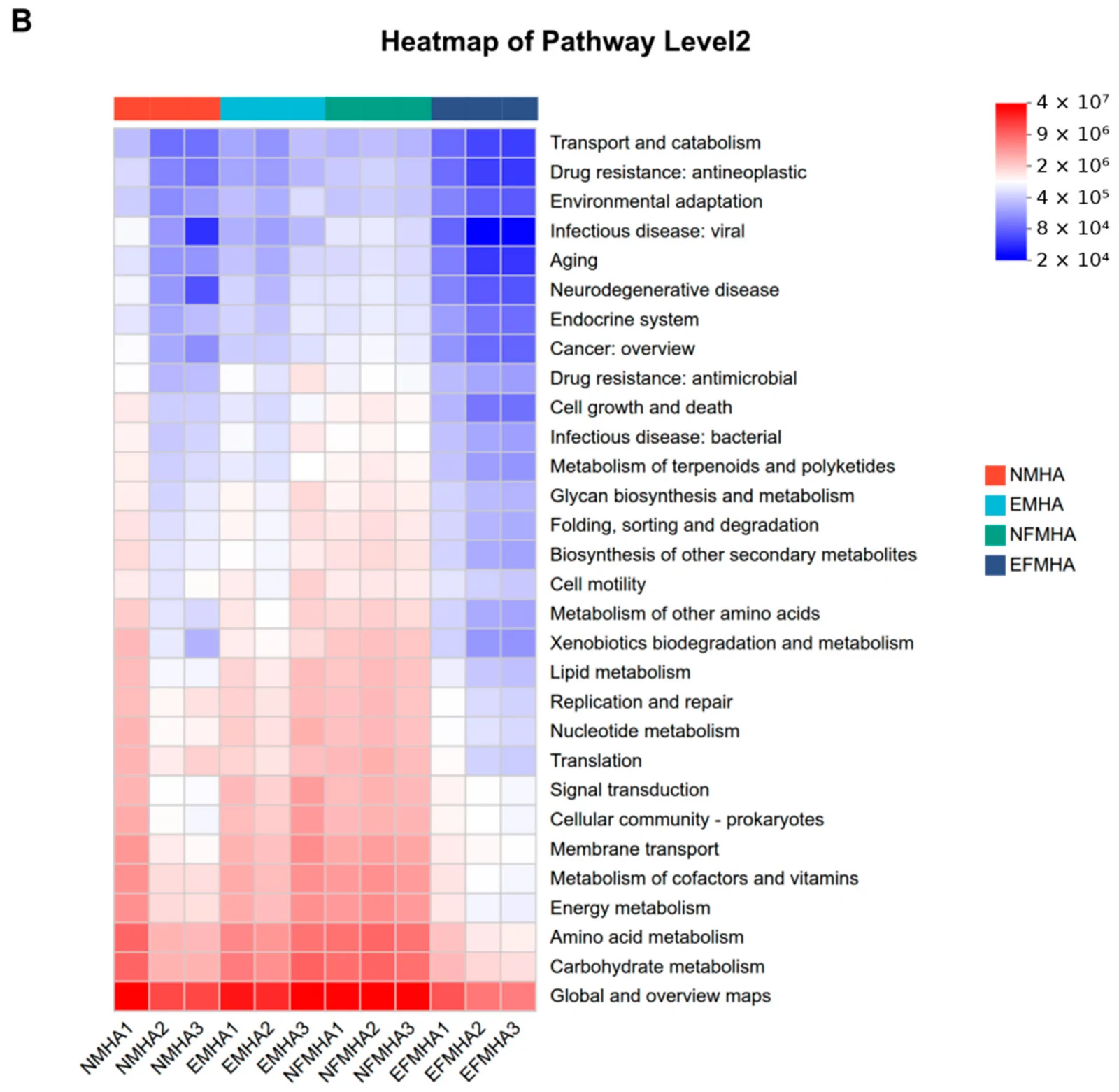

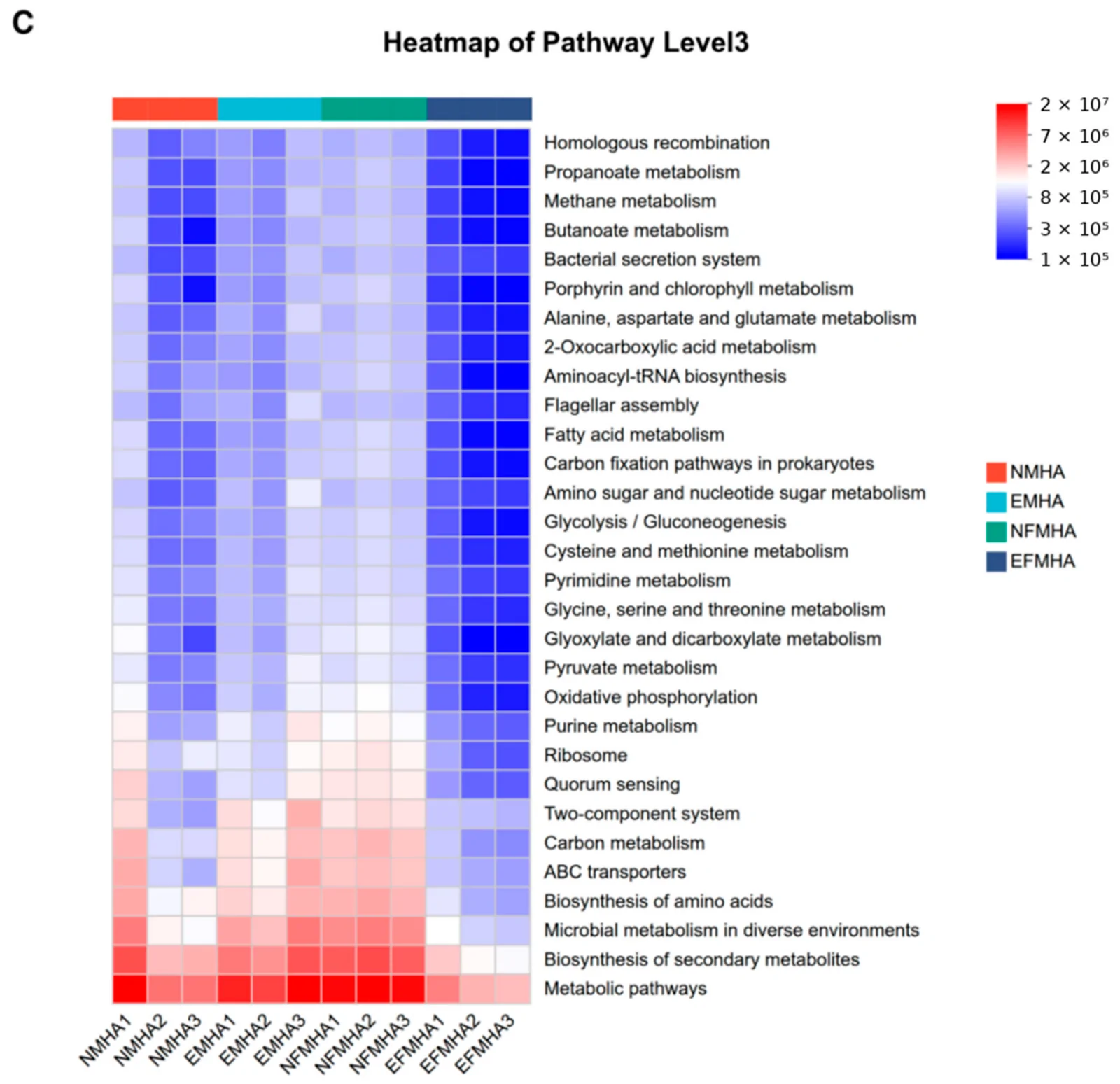
Predicted functional profiles of gut microbiota across the four groups based on PICRUSt2 analysis. (**A**) Heatmap of KEGG Orthology (KO) categories. (**B**) Heatmap of KEGG pathway Level 2 categories. (**C**) Heatmap of KEGG pathway Level 3 categories. In each heatmap, columns represent individual pooled samples and rows represent predicted functional features. The color gradient indicates normalized predicted abundance, with red representing higher values and blue representing lower values. At KEGG Level 2, diseased groups, particularly diseased females, showed higher predicted representation of pathways associated with membrane transport, signal transduction, cell motility, and cellular community–prokaryotes. At KEGG Level 3, differences were observed in the predicted representation of pathways associated with bacterial chemotaxis, flagellar assembly, biofilm formation, two-component systems, ABC transporters, and phosphotransferase systems. These patterns indicate differences in predicted functional potential among groups. Because these profiles were inferred from 16S rRNA gene data using PICRUSt2, they should not be interpreted as direct measurements of microbial gene expression, metabolic activity, or functional capacity.

Diseased females also showed higher predicted representation of several pathways associated with bacterial motility and host interaction. The predicted relative abundance of bacterial chemotaxis was approximately 2.17-fold higher in diseased females, while little change was observed in diseased males. Flagellar assembly showed approximately 1.39-fold higher predicted representation in diseased females but approximately 0.81-fold relative abundance in diseased males compared with their respective healthy groups. The bacterial secretion system and lipopolysaccharide biosynthesis also showed higher predicted representation in diseased females (approximately 1.57-fold and 1.43-fold, respectively). Collectively, these PICRUSt2-derived patterns indicate differences in predicted functional potential and should not be interpreted as direct evidence of changes in microbial gene expression or metabolic activity. These predicted functional profiles are visualized in Figure 8A–C.

## 4. Discussion

### 4.1. Disease-Associated Gut Microbiota Dysbiosis Primarily Manifests as Structural Community Rearrangement

Disease-associated shifts in the gut microbiota in seahorses primarily manifested as significant rearrangements of community structure, rather than a simple decline in diversity. The two-way comparison framework of the four groups and the combined sensitivity analysis (“Normal vs. Diseased”) both showed that diseased individuals consistently exhibited significant separation in β-diversity, while α-diversity only showed a declining trend, and this reduction was primarily concentrated in diseased females. This indicates that, following the onset of disease, the gut microecology is more likely to experience disruptions in its community composition and in the relative abundance of dominant taxa, rather than a synchronous depletion of all microbial lineages.

This observation is in accord with previous findings in seahorses and other marine aquaculture species. Studies showed that the gut microbiota composition in yellow seahorses with enteritis underwent obvious shifts [3,7]. Similarly, the gut microbiota and metabolic profiles exhibited systemic remodeling in a model of *E. piscicida* infection in the big-belly seahorse [9,11]. Analogous phenomena have been observed in studies of acute *Vibrio alginolyticus* infection in half-smooth tongue sole and bacterial infection in *Litopenaeus vannamei*, where significant separation in community composition often preceded, and did not necessarily coincide with, a synchronized decline in richness and evenness indices [28,34]. These results suggest that, under conditions of pathogen infection or inflammatory stress, the early or primary characteristics of gut dysbiosis are more likely characterized by the replacement of dominant taxa, community structural shifts, and niche reallocation, rather than simply a loss of microbes.

### 4.2. Sex-Stratified Disease-Associated Gut Microbiota Changes

The current study showed that disease-associated changes in the gut microbiota differed between males and females during this outbreak. Compared with relatively mild sex-related differences in apparently normal individuals, diseased females showed a more pronounced decline in α-diversity and stronger deviations in β-diversity, together with a community structure dominated by a single predominant taxon. Notably, apparently normal females (NFMHA) possessed the highest richness, phylogenetic breadth, and a compositionally diverse microbiota, indicating relatively high community complexity under apparently normal conditions. In contrast, diseased females showed a marked restructuring of this community. Thus, a stronger disease-associated microbiota shift was observed in females during this particular outbreak, characterized by a transition from a relatively diverse community to a low-diversity, *Vibrio*-dominated state, whereas males showed less pronounced community restructuring and retained a greater proportion of background taxa. However, this outbreak-specific pattern should not be interpreted as evidence of intrinsic female susceptibility. Differences in disease severity, reproductive status, physiological condition, feeding behavior, hormonal state, individual susceptibility, or other unmeasured factors may also have contributed to the observed sex-stratified patterns. Given the limited effective sample size (*n* = 3 pooled biological replicates per group), the Health × Sex interaction detected by the two-way ANOVA should be interpreted cautiously. Nevertheless, the significant Health × Sex interaction detected by PERMANOVA (*p* = 0.0037) provides complementary multivariate support for sex-stratified disease-associated differences in gut microbial community structure.

These findings are consistent with the general trends observed in previous studies on fish and seahorses. Sex-biased distributions of gut microbiota have been documented in hermaphroditic fish and obscure cultured pufferfish [21,22]. Sex differences were also closely linked to reproductive hormone levels, fatty acid composition, and changes in metabolic state in species such as *L. catla* and *Channa striata* [23,24]. From a broader perspective of host–microbe interactions, biological sex and sex-related physiological and immune states have been associated with differences in microbial community composition and host–microbiota interactions [25,35]. Furthermore, studies on reproduction-related microbiomes and environmental antibiotic exposure in *H. abdominalis* revealed high plasticity in mucosal microecology [19,20], suggesting that the gut microbiota of *H. abdominalis* may vary with host physiological status and environmental perturbations.

### 4.3. Vibrio Enrichment Is a Prominent Feature of Disease-Associated Gut Dysbiosis

Among the differential taxa, *Vibrio* showed the most prominent disease-associated enrichment, particularly in diseased females. Its consistent identification in pairwise comparisons and LEfSe analysis, together with the concurrent decline in Shannon diversity and marked community restructuring, suggests that *Vibrio* enrichment was closely associated with the dysbiotic state observed during the mortality outbreak. The gut microbiota of diseased females was characterized by pronounced *Vibrio* dominance, whereas diseased males also showed increased *Vibrio* abundance but retained a greater proportion of background taxa. These patterns suggest that the magnitude of disease-associated *Vibrio* enrichment differed between sexes.

This observation is biologically plausible because *Vibrio* species are common opportunistic pathogens in marine aquaculture animals and are frequently associated with enteritis, tissue damage, and mortality [5,9,36,37]. In seahorses, *V. alginolyticus, V. splendidus*, and *V. tubiashii* have been isolated from diseased individuals, and experimental infection studies have demonstrated the pathogenic potential of some *Vibrio* species [5,6,37]. Therefore, the enrichment of *Vibrio* observed in diseased seahorses, particularly females, is consistent with previous reports of disease-associated *Vibrio* proliferation in marine aquaculture species.

However, the cross-sectional design of the present study does not establish temporal precedence or causality. The observed *Vibrio* enrichment may have contributed to disease-associated gut dysbiosis, but it may also have occurred as a consequence of disease-related changes in the intestinal environment. Therefore, *Vibrio* should be interpreted here as a disease-associated microbial feature rather than a confirmed causal agent. Moreover, 16S rRNA gene sequencing does not provide sufficient resolution to identify the specific *Vibrio* species or strain associated with the observed enrichment.

### 4.4. Dynamics of Background and Concomitant Taxa Should Be Interpreted Within a Niche Rearrangement Framework

Beyond the prominent expansion of *Vibrio*, alterations in other differential taxa warrant attention; however, their biological significance is best interpreted within the framework of community niche rearrangement. Compared with the diseased groups, apparently normal seahorses (especially apparently normal females) retained a richer array of background taxa, including Hyphomicrobiaceae, Microtrichaceae, *Filomicrobium*, *Methylobacterium*, and *Burkholderia-Caballeronia-Paraburkholderia*. While these taxa may not necessarily possess definitive probiotic properties, their co-occurrence suggests that healthy individuals maintained higher community complexity, functional redundancy, and colonization stability in their intestines. Previous studies indicated that seahorse-associated bacterial communities exhibited high intrinsic heterogeneity across different species and habitats [17,18], and factors such as reproductive status and antibiotic exposure further modified the gut and mucosal microecology of *H. abdominalis* [19,20]. These taxa characterized the apparently normal groups in this outbreak, but their ecological roles cannot be established from the present 16S data and they should not be categorized uniformly as beneficial bacteria.

In contrast, increases in certain concomitant taxa in diseased samples more likely reflect disease-driven niche opening and opportunistic colonization. For instance, *Arcobacter* and *Litoreibacter*, which were relatively enriched in diseased males, as well as *Pseudoalteromonas*, which fluctuated upwards in some diseased samples, are better considered as concomitant responder signals during community dysbiosis, rather than being definitively designated as primary pathogens based solely on 16S data. *Pseudoalteromonas*, in particular, exhibits a dual nature in aquatic systems: some strains possess *Vibrio*-antagonistic potential and are thought to participate in microecological defense [30], while other members have been associated with diseases in shellfish [32]. This study suggests that these taxa may be interpreted as co-competitors or secondary colonizers within the dysbiotic niche, rather than definitive causal agents on a par with *Vibrio*.

### 4.5. Predicted Functional Profiles Are Associated with Pathways Related to Colonization and Stress Responses

PICRUSt2 analysis suggested that disease-associated changes in microbial community composition were accompanied by differences in predicted functional potential. In the diseased groups, higher predicted representation was observed for pathways associated with the phosphotransferase system, two-component system, biofilm formation, cationic antimicrobial peptide resistance, and ABC transporters. Diseased females additionally showed higher predicted representation of pathways associated with chemotaxis, flagellar assembly, secretion systems, and lipopolysaccharide/lipid A biosynthesis. These patterns were associated with predicted functional potential related to environmental sensing, colonization, motility, and stress responses. However, because these functions were inferred from 16S rRNA profiles, they should not be interpreted as direct evidence of enhanced microbial activity or virulence.

This trend is largely consistent with the results of previous studies. Studies on *E. piscicida* infection in *H. abdominalis* and acute *V. alginolyticus* infection in half-smooth tongue sole both demonstrated that the gut microbiota could remodel towards directions associated with host interaction and virulence [9,11,28]. Reviews on marine vibriosis also highlighted motility, adhesion/biofilm formation, signal regulation, and tolerance mechanisms as critical components of *Vibrio* colonization and pathogenesis [26,27]. Thus, the predicted functional profiles were broadly consistent with pathways previously associated with environmental sensing, motility, colonization, and stress responses. Direct functional validation will be required to determine whether these inferred differences correspond to actual changes in microbial activity.

### 4.6. Study Limitations

This study has several limitations. First, although 36 individual seahorses were sampled, the pooling strategy resulted in only 12 microbiome experimental units, with three pooled biological replicates per group. Pooling before DNA extraction inevitably averaged interindividual variation and prevented assessment of individual-level microbial heterogeneity. The small number of pooled biological replicates per group (*n* = 3) also limited the statistical power of the two-way ANOVA, particularly for detecting and interpreting interaction effects. Therefore, the ANOVA results should be considered preliminary, and the overall findings should be interpreted primarily as group-level patterns rather than individual-level responses. Because intestinal contents were pooled before DNA extraction and sequencing, individual-level 16S rRNA sequencing data were not generated and therefore cannot be provided as supplementary material.

In addition, no extraction blanks, PCR-negative controls, or mock-community positive controls were included, limiting the assessment of potential background contamination and technical bias. The cross-sectional design also precludes determination of the temporal sequence or causality of the observed microbiota changes. All animals originated from a single aquaculture facility, a single aquaculture tank, and a single naturally occurring mortality outbreak; therefore, independent tank- or facility-level environmental variation could not be evaluated, which limits the generalizability of the findings. Because apparently normal and diseased seahorses were co-housed and sampled during the same outbreak, apparently normal individuals may have been exposed to the same outbreak-associated factors while remaining asymptomatic at the time of sampling, and a pre-symptomatic state cannot be excluded. Potential differences in disease severity, reproductive status, physiological condition, feeding behavior, hormonal state, and other unmeasured host- or environment-related factors may also have contributed to the observed sex-stratified patterns.

Moreover, 16S rRNA gene sequencing lacks sufficient taxonomic resolution to identify the specific *Vibrio* species or strain associated with the observed enrichment. Because pathogen isolation, species-specific molecular confirmation, histopathological validation, and challenge experiments were not performed, the enrichment of *Vibrio* should be interpreted as a disease-associated microbial feature rather than evidence of a confirmed causal pathogen. Finally, PICRUSt2-derived functional profiles were inferred from 16S rRNA gene data and therefore represent predicted functional potential rather than experimentally measured microbial gene expression, metabolic activity, or functional capacity.

Accordingly, the present study should be regarded as an exploratory, outbreak-associated microbiome study, and the observed patterns require validation in larger independent cohorts using individual-level sequencing, longitudinal sampling, appropriate laboratory controls, and pathogen-specific validation.

## 5. Conclusions

Based on a two-way factorial design, this exploratory outbreak-associated study identified sex-stratified patterns of gut microbiota dysbiosis in cultured *H. abdominalis* during a naturally occurring mortality event. Diseased female seahorses showed pronounced reductions in microbial diversity and reduced community complexity, accompanied by marked dominance of *Vibrio* (relative abundance approaching 87%). In contrast, diseased males showed less pronounced community restructuring and retained greater microbial diversity and a larger proportion of background taxa. Two-way analyses and PERMANOVA provided statistical evidence suggesting a Health × Sex interaction in gut microbial patterns. However, given the limited effective biological replication (*n* = 3 pooled samples per group), this interaction effect should be regarded as preliminary. These findings suggest that host sex should be considered when evaluating disease-associated gut microbiota patterns in seahorse aquaculture. The pronounced enrichment of *Vibrio* observed in diseased females may represent a candidate disease-associated microbial feature for future validation. Larger independent and longitudinal studies using individual-level sequencing and pathogen-specific validation will be required to confirm these sex-stratified patterns and determine their potential value for future disease surveillance. 

## Figures and Tables

**Table 1:** Experimental grouping and sample size.

Group	Sex	Health Status	Number of Individuals	Number of Pooled Samples (*n*)
NMHA	Male	Normal	9	3
EMHA	Male	Diseased	9	3
NFMHA	Female	Normal	9	3
EFMHA	Female	Diseased	9	3

**Table 2:** Two-way ANOVA results for α-diversity indices.

Metric	Factor	F	*p*	Partial η^2^
Observed ASVs	Health	7.091	0.0287	0.470
Observed ASVs	Sex	0.328	0.5824	0.039
Observed ASVs	Health × Sex	7.242	0.0274	0.475
Faith’s PD	Health	1.495	0.2563	0.157
Faith’s PD	Sex	0.446	0.5230	0.053
Faith’s PD	Health × Sex	1.586	0.2434	0.165
Shannon	Health	7.158	0.0281	0.472
Shannon	Sex	0.121	0.7364	0.015
Shannon	Health × Sex	8.582	0.0190	0.518
Simpson	Health	7.169	0.0280	0.473
Simpson	Sex	2.546	0.1492	0.241
Simpson	Health × Sex	9.449	0.0153	0.542

**Table 3:** Two-way PERMANOVA results for β-diversity based on the Bray–Curtis distance.

PERMANOVA	F	*p*	R
Health	2.299	0.0252	0.154
Sex	1.436	0.2026	0.096
Health × Sex	3.219	0.0037	0.215

## Data Availability

The raw 16S rRNA gene amplicon sequencing data generated in this study have been deposited in the NCBI Sequence Read Archive (SRA) under BioProject accession number PRJNA1497640 and are publicly available at https://www.ncbi.nlm.nih.gov/sra/PRJNA1497640 (accessed on 14 August 2026). Other original contributions presented in this study are included in the article. Further inquiries can be directed to the corresponding authors.
